# Structural Design of Ceramic Membranes to Mitigate Fouling in Membrane Bioreactors

**DOI:** 10.3390/membranes16090297

**Published:** 2026-09-10

**Authors:** Boyang Yu, Chao Fan, Tuo Sun

**Affiliations:** 1 Future Water Laboratory, Innovation Center of Yangtze River Delta, Zhejiang University, Jiaxing 314100, China; 2 CCCC First Harbor Consultants Co., Ltd., Tianjin 300461, China; fc13785365035@126.com; 3 Civil Aviation University of China, Tianjin 300461, China; sunt@cauc.edu.cn

**Keywords:** hollow flat-sheet ceramic membrane, membrane bioreactor, biofouling, symmetric/asymmetric membrane architecture, microbial community, EPS

## Abstract

Despite the robust mechanical and chemical stability that make hollow flat-sheet ceramic membranes highly attractive for membrane bioreactors (MBRs), the fundamental relationship between their structural design, specifically pore size and structural symmetry, and biological fouling behavior remains elusive. To decouple the effects of membrane architecture on fouling mechanisms, a series of symmetric and asymmetric hollow flat-sheet alumina membranes were systematically engineered. Symmetric architectures with tunable pore sizes were fabricated by controlling aggregate particle sizes, whereas asymmetric counterparts featuring distinct separation layer thicknesses were developed via a tailored dip-coating process. Long-term operational evaluations treating municipal wastewater uncovered a counterintuitive phenomenon. Asymmetric membranes, despite yielding superior retention, experienced markedly accelerated transmembrane pressure evolution and severe cake layer fouling compared to the symmetric supports. Resistance-in-series analysis coupled with classical filtration models demonstrated that thicker separation layers and larger pore sizes were associated with shifts in the dominant fouling mechanism toward rapid and dense cake layer formation, which significantly exacerbated irreversible biological fouling. Furthermore, advanced spectroscopic and high-throughput sequencing techniques revealed that structurally complex asymmetric layers were associated with shifts in extracellular polymeric substances and specific fouling-associated bacterial phyla at the membrane interface. Ultimately, these findings underscore the necessity of architectural optimization to mitigate biofouling and prolong the operational lifespan of ceramic membranes, highlighting the sustainable advantages of symmetric structures.

## 1. Introduction

MBR technology is a primary approach for wastewater treatment, effectively combining biological degradation with physical membrane filtration [1,2]. This integration ensures complete retention of biomass, yielding excellent effluent quality and enabling operation at elevated mixed liquor suspended solids (MLSS) concentrations within a compact footprint [3]. Although polymeric membranes are widely utilized in MBRs, they suffer from limited chemical resistance and shortened lifespans under harsh cleaning conditions [4,5]. Consequently, ceramic membranes provide a robust alternative, distinguished by their high mechanical strength, excellent thermal stability, and superior chemical resilience [6,7]. Among these, hollow flat-sheet ceramic membranes have garnered specific attention, as they combine the high packing density of hollow fibers with the favorable hydrodynamics of flat-sheet geometries, facilitating effective aeration scouring and stable long-term operation [8].

Despite these operational advantages, membrane fouling remains the principal technical obstacle limiting the broader application of ceramic MBRs [9]. In biological wastewater treatment, fouling is primarily driven by the accumulation of microbial flocs, soluble microbial products (SMP), and extracellular polymeric substances (EPS) on the membrane surface and within its porous structure [10]. These foulants interact with the membrane to form a highly compressible cake layer and induce internal pore blockage, steadily increasing the transmembrane pressure (TMP) and diminishing permeation flux [11,12]. Current strategies to control fouling rely heavily on periodic physical cleaning to remove loosely attached foulants, supplemented by rigorous chemical cleaning to dissolve irreversible foulants [13,14]. However, frequent chemical interventions inflate operational costs, generate secondary chemical waste, and eventually degrade membrane performance. Therefore, modifying the intrinsic structural design of the membrane to proactively delay fouling onset has emerged as a crucial focus for optimization [15]. Notably, the attachment of bacterial cells, especially large-cell-size and filamentous microorganisms, to the membrane surface, rather than their presence in the bulk liquid, is a primary driver of biofilm formation and cake layer development. This inherent cell–surface interaction presents a fundamental limitation to MBR applications. Pretreatment steps such as coagulation and filtration have therefore been explored as complementary strategies to reduce particulate and colloidal foulant loading, and their integration with optimized membrane design represents a promising direction for comprehensive fouling control.

Existing literature on membrane structural design indicates that parameters such as pore size, pore size distribution, and cross-sectional symmetry significantly influence filtration performance and fouling behaviors [16,17]. Symmetrical membranes possess a uniform pore structure throughout their cross-section, whereas asymmetrical membranes feature dense separation layers deposited on a macroporous support to minimize hydraulic resistance while maintaining high retention [18]. Previous studies on pore size report conflicting observations: some suggest that smaller pores accelerate the formation of a dense cake layer, while others argue that larger pores allow foulants to penetrate deeper, causing severe, physically irreversible internal pore blockage [19,20]. Furthermore, while asymmetric ceramic membranes are favored in various industrial applications for their high initial permeability, their fouling mechanisms in the complex biological suspensions of MBRs have not been systematically compared with symmetrical structures [21]. Specifically, there is a lack of research decoupling how different membrane architectures dictate the dynamic accumulation of organic foulants and the specific microbial composition of the resulting biological cake layer. Here, “decoupling” means independently varying the pore size of symmetric membranes and the separation-layer thickness of asymmetric membranes while holding other fabrication variables constant, as described in Section 2.1.

To address this gap, this study investigates how membrane structural parameters affect biofouling characteristics in an MBR treating municipal wastewater. A series of symmetric hollow flat-sheet alumina membranes with varying pore sizes were fabricated via extrusion molding. Subsequently, asymmetric alumina membranes with varying separation layer thicknesses were prepared on the optimized symmetric support using a controlled dip-coating method [22]. These two distinct categories of structural designs were evaluated in a continuously operating MBR system. Specifically, the research objectives encompass evaluating the macroscopic fouling rates and quantifying the distribution of reversible and irreversible fouling resistances to elucidate the governing filtration mechanisms. Furthermore, this study seeks to characterize the interfacial organic foulant composition and microbial community structures within the cake layers across different membrane architectures to better understand their associations with biofouling behavior. We note that irreversible fouling resistance, operationally defined as resistance remaining after physical cleaning, is predominantly biological in the MBR context but may also include non-biological components such as organic adsorption and inorganic deposition.

Ultimately, this research clarifies the relationship between hollow flat-sheet ceramic membrane structure and biofouling mechanisms. By comprehensively comparing the fouling resistance distributions and the microbiological properties of the foulant layers across symmetric and asymmetric architectures, this study provides critical theoretical guidance and practical engineering references for optimizing ceramic membrane manufacturing. The novelty of this study lies in the systematic decoupling and comparative analysis of symmetric pore-size variation and asymmetric separation-layer thickness variation, and their differential impacts on fouling resistance, organic foulant composition, and interfacial microbial communities, providing theoretical guidance for ceramic membrane optimization. These insights will facilitate the development of highly fouling-resistant membrane modules for sustainable MBR applications. Building on these findings, key research directions include real wastewater validation, long-term chemical cleaning cycling, integration with pretreatment strategies, and functional metagenomic/transcriptomic analysis for microbial causality.

## 2. Materials and Methods

### 2.1. Membrane Preparation

**Figure 1:**
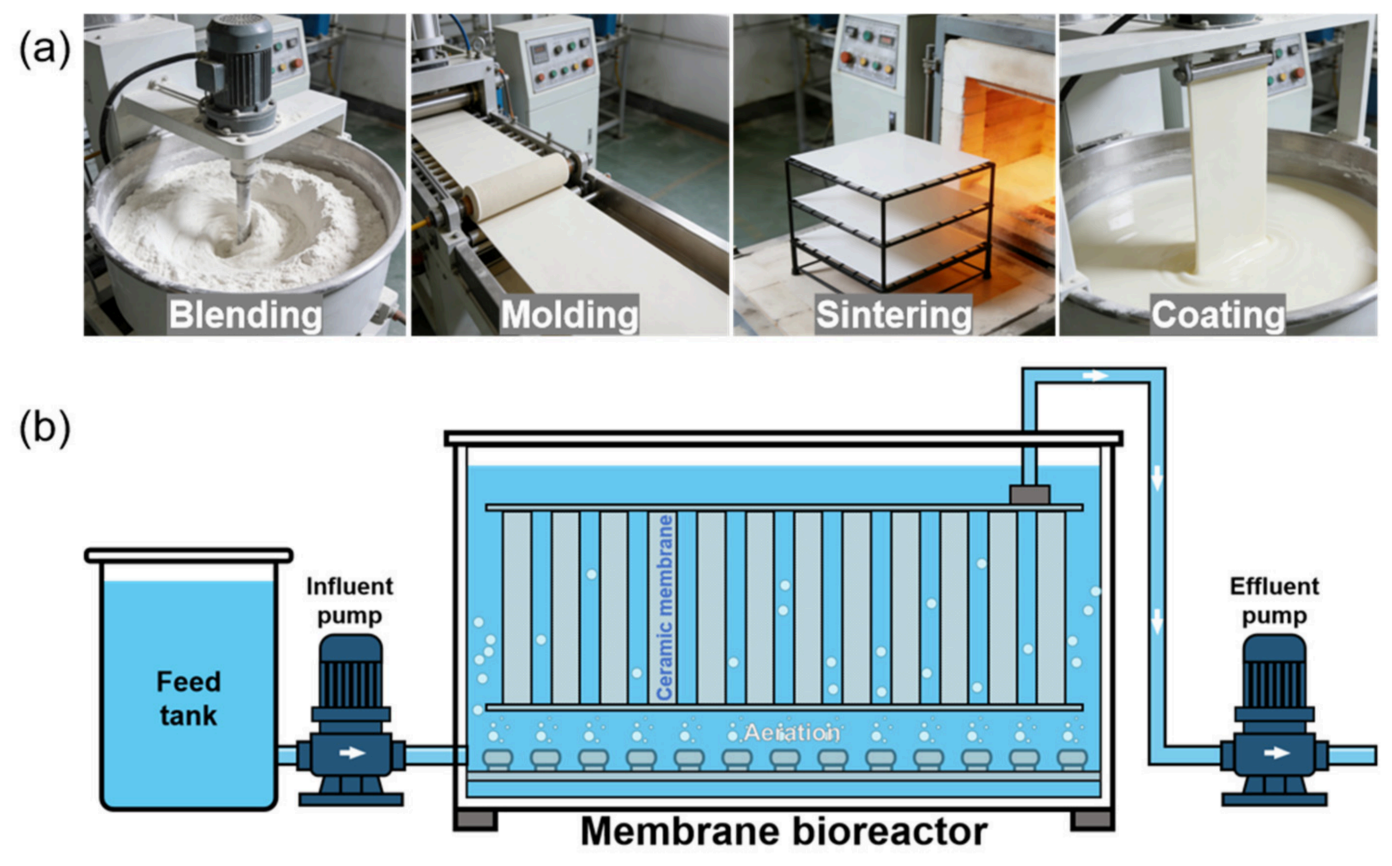
(**a**) Schematic Diagram of the Hollow Flat Ceramic Membrane Production Process. (**b**) Schematic Diagram of Ceramic MBR.

To systematically investigate the effect of membrane architecture on fouling behaviors, both symmetric and asymmetric hollow flat-sheet ceramic membranes were prepared. Symmetric membranes were synthesized via an extrusion molding process. Briefly, α-Al_2_O_3_ powders serving as the primary aggregate were uniformly blended with specific ratios of organic binders, pore-forming agents, lubricants, and solvents to yield a homogeneous, plastic paste. Following vacuum pugging and an aging period to enhance plasticity, the paste was extruded through a customized die to form the multi-channel hollow flat-sheet geometry. The green bodies were then dried at room temperature and subjected to high-temperature sintering to obtain rigid, symmetric ceramic membranes. Asymmetric membranes were subsequently developed utilizing the aforementioned symmetric membranes as macroporous supports. A highly stable coating suspension was prepared by ball-milling finer α-Al_2_O_3_ particles with specific dispersants and binders. The asymmetric separation layer was deposited onto the support surface using a meticulously controlled dip-coating method, wherein the support was immersed vertically into the suspension and withdrawn at a constant velocity. After a strict temperature- and humidity-controlled drying process to prevent micro-cracking, the membranes underwent a secondary thermal sintering step to consolidate the dense, continuous active separation layer. The preparation process is shown in Figure 1a. Specific procedures for membrane preparation are documented in Text S1, and the particle size distribution of the α-Al_2_O_3_ powder is shown in Figure S1.

### 2.2. Membrane Characterization

The intrinsic physicochemical and structural properties of the fabricated symmetric and asymmetric membranes were systematically characterized to establish a comprehensive baseline prior to the MBR fouling experiments. Surface and cross-sectional morphologies were observed using a field-emission scanning electron microscope (FESEM, SU8010, Hitachi, Tokyo, Japan). The thickness of the asymmetric separation layers and the surface pore size distributions were quantitatively determined from the FESEM images using ImageJ software (ImageJ1). The pure water flux (PWF) of the distinct membrane architectures was evaluated using a laboratory-scale dead-end filtration setup. The tests were conducted under a constant transmembrane pressure (0.1 bar) at room temperature to quantify the intrinsic hydraulic resistance induced by the asymmetric separation layers. To ensure the structural integrity of the membranes under harsh cleaning conditions, their chemical stability was rigorously tested. The membrane samples were completely immersed in strong acidic (1 M HCl, pH ≈ 1) and alkaline (1 M NaOH, pH ≈ 13) solutions for 15 days. The corrosion resistance was subsequently evaluated by calculating the precise mass loss ratio of the dried samples before and after the chemical exposure.

### 2.3. MBR Setup and Operational Parameters

The fouling behaviors of the ceramic membranes were evaluated in a laboratory-scale (6 L) submerged MBR system treating synthetic municipal wastewater (Figure 1b). The composition and preparation methods for the simulated water are listed in Table S1. The reactor was inoculated with activated sludge from a local municipal wastewater treatment plant (Tianjin, China) with an initial MLSS of approximately 3 g·L^−1^. Prior to the experiments, the sludge was pre-aerated for 24 h. To ensure comparability, membrane modules with distinct architectural features were operated in parallel. The MBR was driven by peristaltic pumps (BT100-2J, Longer Precision Pump, China) under a constant-flux mode (15 L·m^−2^·h^−1^) for 60 days. Four reactors housed the same membrane module of identical effective membrane area, and each architecture was evaluated in its own independent biological reactor. To ensure identical biological conditions, all four reactors were fed with the same synthetic municipal wastewater and inoculated with the same batch of activated sludge. To proactively manage fouling, an intermittent suction cycle was implemented (8 min of filtration followed by 2 min of relaxation). Continuous aeration was supplied via bottom diffusers to maintain a dissolved oxygen (DO) concentration above 2 mg·L^−1^ and generate hydrodynamic shear flow to minimize dead zones and scour the membrane surfaces. Throughout the operation, the mixed liquor suspended solids (MLSS) concentration, hydraulic retention time (HRT), and solids retention time (SRT) were controlled at 8 g·L^−1^, 8 h, and 20 d, respectively. Influent and effluent water quality indicators (COD, NH_4_^+^-N, and TP) were routinely monitored using standard spectrophotometric methods to assess the biological treatment efficacy.

### 2.4. Analytical Methods

Macroscopic fouling evolution was continuously monitored by recording the TMP using high-precision pressure transducers. To elucidate the governing filtration mechanisms, the initial constant-flux filtration data were fitted to the Hermia model [23] (Text S2) to distinguish among complete blocking, standard blocking, intermediate blocking, and cake filtration mechanisms. Furthermore, based on Darcy’s law [24] (Text S2 and Table S2), the total resistance, intrinsic membrane resistance, reversible fouling resistance, and irreversible fouling resistance were systematically quantified following specific physical rinsing protocols.

(1) $$\frac{d^{2}t}{dV^{2}}=k{\left(\frac{dt}{dV}\right)}^{n}$$

(2) $$R_{t}=R_{m}+R_{pr}+R_{cr}+R_{ir}=\frac{\Delta P}{\mu \cdot J}$$

where t is the filtration time, V is the cumulative permeate volume, k is the fouling rate constant (units depend on n), and n is the fouling mechanism index; R_t_ is the total filtration resistance, R_m_ is the intrinsic membrane resistance, R_pr_ is the pore-blocking resistance, R_cr_ is the cake layer resistance, Rir is the irreversible fouling resistance, ΔP is the transmembrane pressure, μ is the dynamic viscosity of the permeate, and J is the permeate flux. To decode the microscopic mechanisms governing membrane fouling, a comprehensive analysis of the accumulated foulants was conducted post-operation. The morphology of the biological cake layer was directly observed via SEM (SIGMA 500, Carl Zeiss, Darmstadt, Germany). The foulants were then carefully harvested and centrifuged to extract extracellular polymeric substances (EPS), and the separation methods are recorded in Text S3. The composition and distribution of the organic foulants were qualitatively characterized using three-dimensional excitation–emission matrix (EEM) fluorescence spectroscopy (F-7000, Hitachi, Japan). The absolute concentrations of proteins and polysaccharides within the fouling layer were quantitatively determined using a modified Lowry method and the phenol-sulfuric acid method, respectively [25,26]. Finally, to elucidate the biological fouling mechanisms at the microbiological level, the microbial community structure within the cake layers was analyzed using high-throughput 16S rRNA gene amplicon sequencing, decoding how specific membrane architectures selectively enrich distinct microbial populations at the interface. Quantitative measurements (EPS protein and polysaccharide concentrations, TMP growth rates) were performed in at least technical triplicate, and data are presented as mean ± standard deviation (SD). One-way analysis of variance (ANOVA) followed by Tukey’s post-hoc test was employed to evaluate statistical significance, with a significance threshold of *p* < 0.05. Due to limited biomass recoverable from individual membrane modules, microbial DNA was extracted from pooled duplicate membrane samples per architecture for 16S rRNA sequencing.

## 3. Results and Discussions

### 3.1. Properties of the Membranes

**Figure 2:**
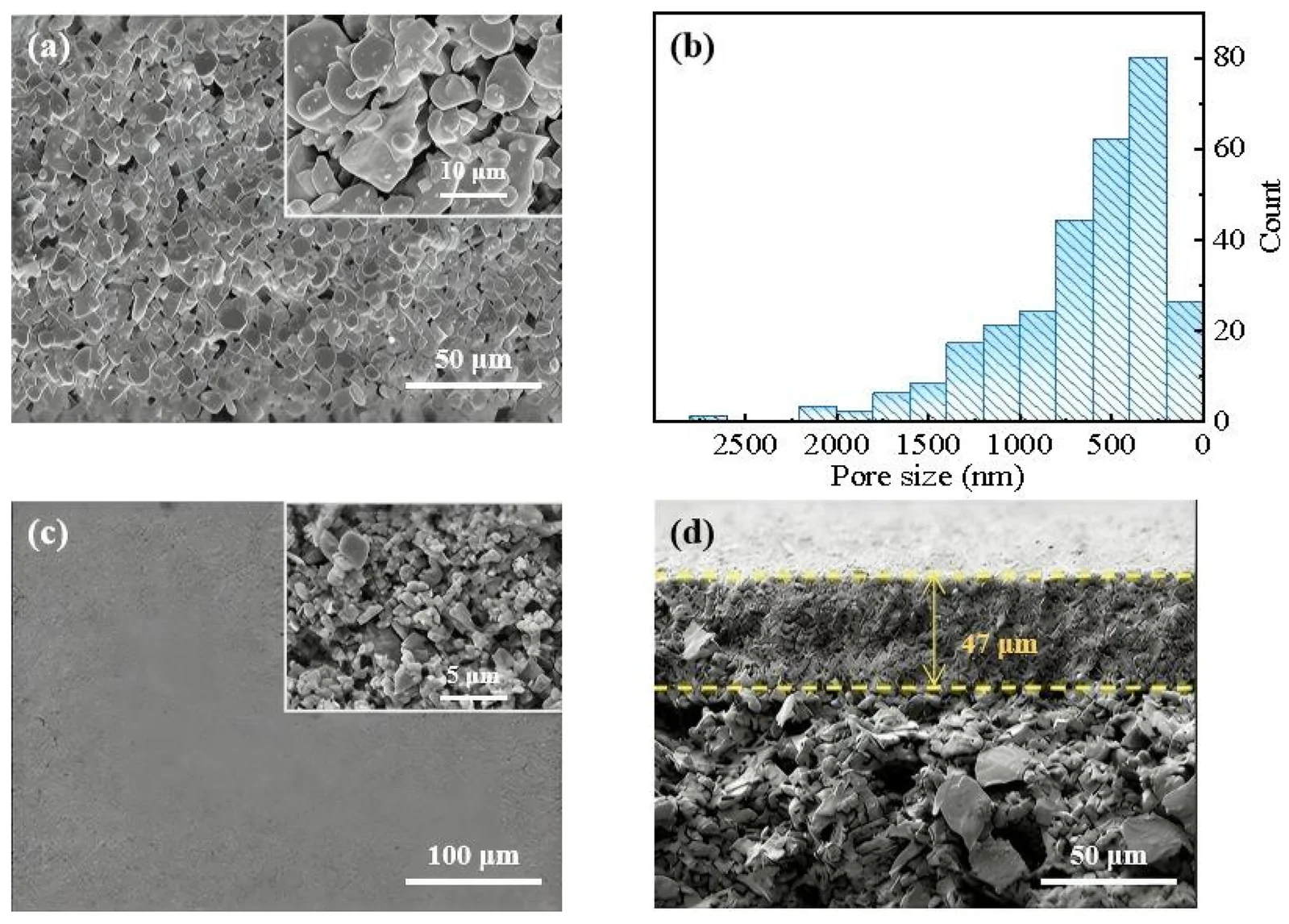
(**a**) Surface SEM image of the symmetric membrane, with the inset showing the high-magnification morphology. (**b**) Pore size distribution histogram of the symmetric membrane. (**c**) Surface SEM image of the asymmetric membrane, with the inset showing the high-magnification morphology. (**d**) Cross-sectional SEM image of the asymmetric membrane; the yellow dashed lines indicate the thickness of the separation layer.

To elucidate the impact of architectural design on filtration performance, the microstructures of the fabricated hollow flat-sheet ceramic membranes were systematically characterized. Compositionally, the fundamental matrix of the fabricated membranes consists of high-purity α-Al_2_O_3_ (Figure S2). Figure 2 and Figure S3 present the cross-sectional and surface morphologies of the symmetric and asymmetric membranes. The symmetric membrane, functioning as the macroporous support, exhibited a homogeneous and uniform porous matrix generated by the extrusion and sintering of 8 μm α-Al_2_O_3_ aggregates. In contrast, the asymmetric membranes displayed a distinctive hierarchical structure, characterized by a dense, continuous separation layer firmly bonded to the underlying macroporous support. By adjusting the solid content of the dip-coating slurry, the thickness of this separation layer was precisely tailored to 47 μm, 97 μm, and 153 μm, demonstrating the successful fabrication of structurally intact asymmetric architectures without visible interfacial delamination or macroscopic surface defects.

**Figure 3:**
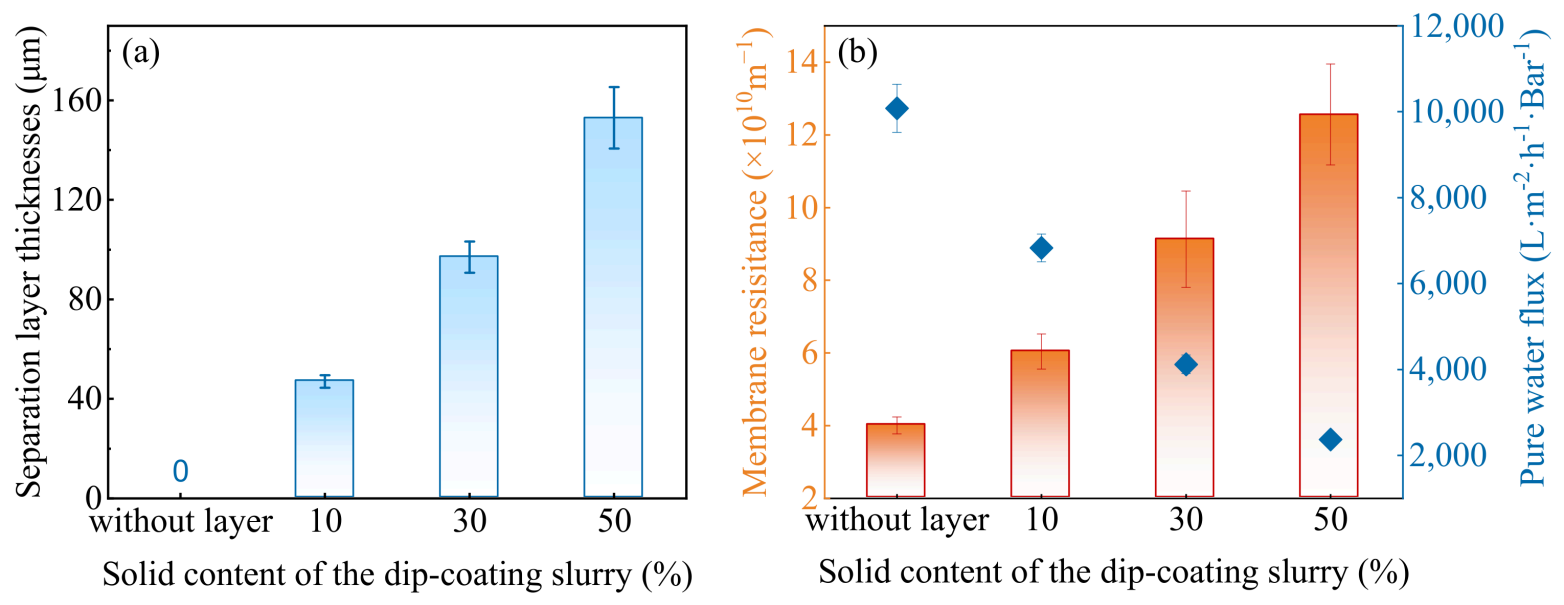
(**a**) Effect of the solid content of the dip-coating slurry on the separation layer thickness. (**b**) Membrane resistance and pure water flux at different solid contents of the dip-coating slurry.

Additionally, all fabricated membranes exhibited exceptional chemical stability with negligible weight loss (<3%) after 15 days of harsh acid/alkali exposure, and the deposition of the separation layer significantly reduced the surface roughness (Figures S4 and S5). The structural modifications fundamentally altered the intrinsic permeability and pore size distribution of the membranes (Figure 3). The symmetric support membrane possessed an average pore size of 0.64 μm, delivering a high pure water flux of 10,800 L·m^−2^·h^−1^·bar^−1^. The deposition of the asymmetric separation layer (comprising finer 1 μm aggregates) significantly narrowed the surface pore size to 0.23 μm, theoretically enhancing the physical retention capabilities. However, this architectural refinement introduced a profound permeability trade-off [27]. The addition of the dense separation layer substantially amplified the intrinsic hydraulic resistance of the membrane. Consequently, the pure water flux exhibited a sharp, thickness-dependent decline, dropping to 6600, 4000, and 2370 L·m^−2^·h^−1^·bar^−1^, respectively. This significant variation in initial hydraulic properties and surface pore dimensions serves as a critical baseline for interpreting the subsequent biofouling dynamics and foulant accumulation behaviors observed during continuous MBR operation.

### 3.2. MBR Operational Performance

**Figure 4:**
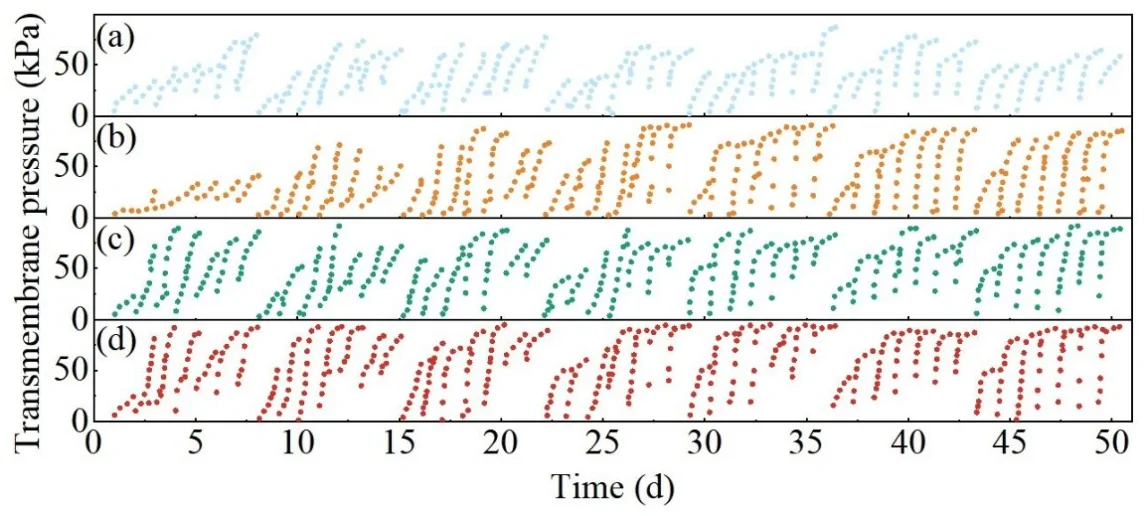
(**a**–**d**) Transmembrane pressure (TMP) variations during continuous operation for the membranes: (**a**) without layer and prepared with dip-coating slurry solid contents of (**b**) 10%, (**c**) 30%, and (**d**) 50%.

Continuous tracking of the transmembrane pressure (TMP) evolution during the long-term MBR operation elucidated the macroscopic fouling propensity of the distinct membrane architectures. Notably, all membrane configurations maintained excellent and stable biological treatment efficacy throughout the continuous operation, securing highly consistent removal efficiencies for organic carbon and nutrients (Figure S6). With the biological performance validated, Figure 4a–d illustrates the dynamic TMP profiles under a constant-flux mode. Initially, all membranes experienced a rapid TMP increase, primarily attributed to the immediate adsorption of soluble microbial products (SMP) and extracellular polymeric substances (EPS) onto the ceramic surfaces before a stable dynamic equilibrium could be established [28,29]. However, as filtration progressed, a counterintuitive divergence in fouling kinetics emerged. The symmetric membrane demonstrated a remarkably lower and more stable fouling rate compared to the asymmetric membranes. Within the asymmetric series, the TMP growth rate accelerated drastically with increasing separation layer thickness. Parallel investigations into the symmetric configurations further revealed a pore-size dependency: membranes with excessively large pores suffered from rapid fouling due to the deep penetration of bio-flocs causing internal blockage, indicating that a symmetric architecture provides the optimal balance for maintaining hydraulic sustainability.

**Figure 5:**
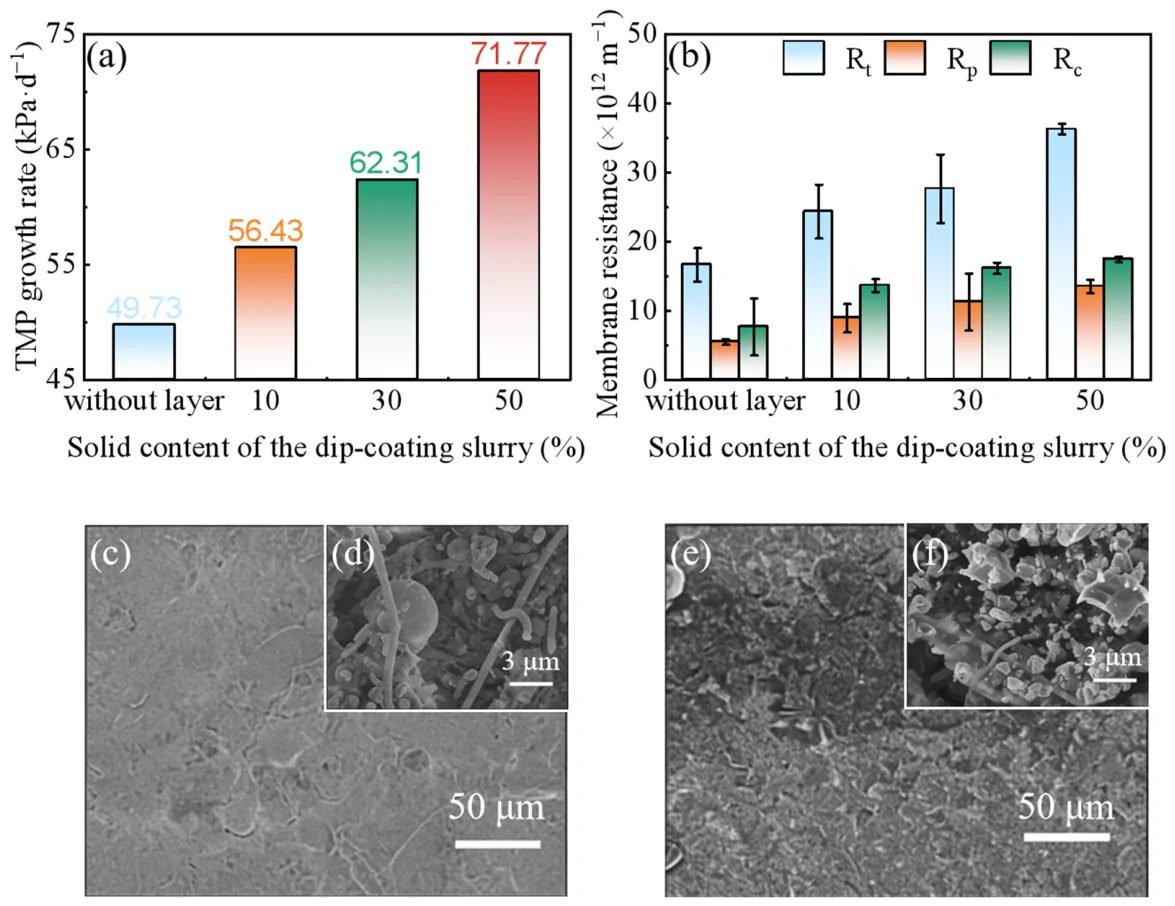
(**a**) TMP growth rates of the membranes with varying solid contents. (**b**) Distribution of membrane fouling resistances. (**c**–**f**) SEM images of the fouled membrane surfaces: (**c**,**d**) membrane without layer and (**e**,**f**) membrane with 30% solid content.

Quantitative analysis of the terminal resistance distribution (Figure 5a,b) reveals the distinct mechanisms driving these divergent TMP trajectories. The asymmetric architectures exhibited a fundamentally different fouling nature compared to the symmetric counterpart. For the symmetric membrane, physically reversible fouling constituted a substantial portion of the total resistance, indicating that the deposited foulants were relatively loosely attached. In stark contrast, the rapid failure of the asymmetric membranes was overwhelmingly dictated by a surge in irreversible biological fouling, compounded by their inherently high intrinsic resistance. This quantitative shift demonstrates that the dense separation layers of the asymmetric membranes severely restrict permeation pathways, creating an elevated local hydrodynamic drag. This concentrated drag forces the macromolecular biological foulants to intensely accumulate and compact precisely at the membrane–water interface. Consequently, routine physical aeration scouring becomes highly ineffective, fundamentally shifting the fouling mechanism toward severe, physically irreversible cake layer filtration. This terminal fouling state was further mathematically corroborated by the classical Hermia filtration model (Table S3), which identified cake layer formation as the dominant mechanism governing the resistance evolution across all samples [30,31]. It is important to distinguish between the initial and terminal filtration stages: during the early stage of filtration, the symmetric membrane exhibited a better fit to the complete pore blocking model, consistent with its larger surface pores allowing initial internal pore penetration, whereas the asymmetric membranes fit the cake filtration model better due to their denser separation layers that immediately promote surface deposition. However, as filtration progresses to the terminal stage, all membrane configurations eventually develop a surface cake layer, and cake filtration becomes the dominant mechanism governing the terminal resistance evolution across all samples. The key difference lies in the rate and extent of cake layer development, which is significantly accelerated for the asymmetric membranes.

Direct morphological evidence of this phenomenon was captured through scanning electron microscopy (SEM) analysis of the fouled membrane surfaces (Figure 5c–f). The visual disparities in the biological cake layers perfectly corroborate the mathematical resistance models. The fouled surface of the symmetric membrane exhibited a relatively heterogeneous and looser foulant deposition, which aligns with its higher proportion of reversible resistance that can be easily mitigated by aeration shear. Conversely, the surfaces of the asymmetric membranes were entirely obscured by a highly dense, uniform, and heavily compacted biological cake layer. The thickness and structural density of this interfacial cake layer visibly intensified on the asymmetric variant with the thickest separation layer. This evidence confirms that while structural refinement via asymmetric separation layers enhances initial physical retention, it unintentionally creates a hydrodynamic bottleneck in complex biological suspensions, thereby exacerbating the rapid accumulation and extreme compaction of bio-foulants. It is important to note that pore structure, surface roughness, and intrinsic hydraulic resistance collectively contribute to the observed fouling resistance, albeit through distinct mechanisms: pore structure governs the initial filtration mode and susceptibility to internal blockage, surface roughness influences initial foulant adhesion, and the elevated hydraulic resistance of asymmetric layers intensifies local permeation drag that drives cake layer compaction. In the present study, the dominant factor differentiating fouling behavior across architectures is the hydraulic resistance-induced local permeation drag, which forces macromolecular biological foulants to intensely accumulate and compact precisely at the membrane–water interface.

### 3.3. Interfacial Organic Foulants

**Figure 6:**
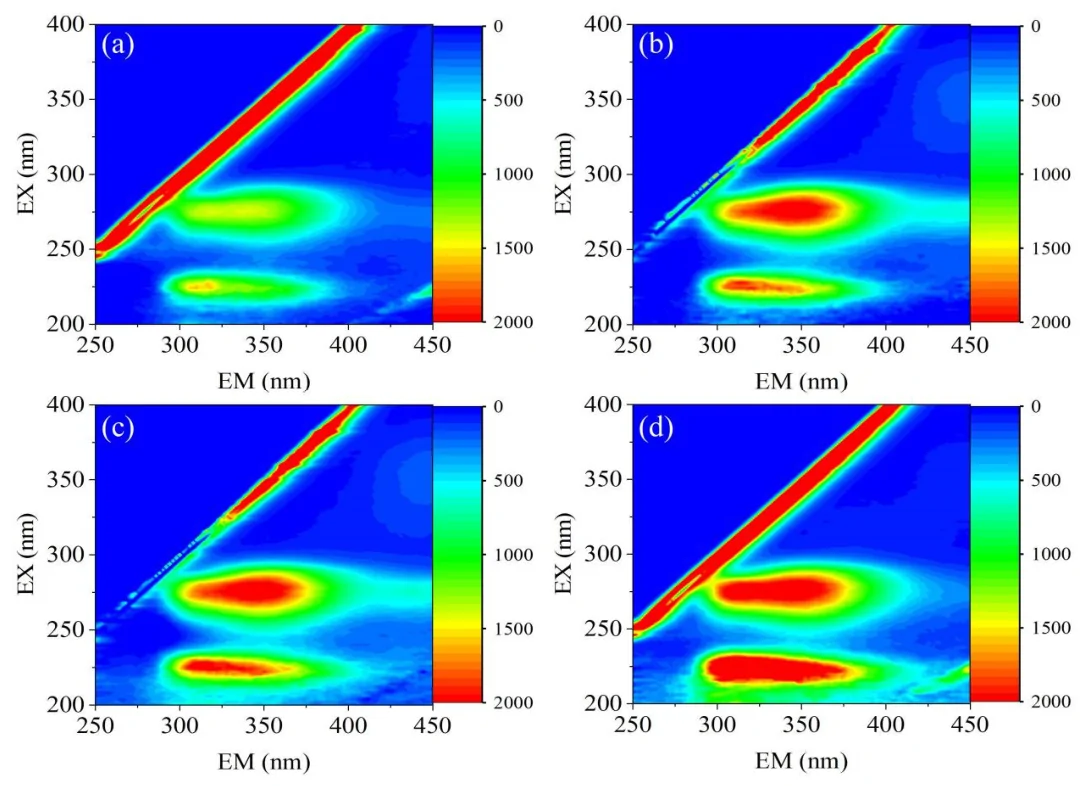
Three-dimensional excitation–emission matrix (3D-EEM) fluorescence spectra of the foulants extracted from the surfaces of different membranes after continuous MBR operation: (**a**) membrane without layer; membranes with dip-coating slurry solid contents of (**b**) 10%, (**c**) 30%, and (**d**) 50%.

Having established that the dense separation layers of the asymmetric architectures trigger severe and irreversible physical cake layer formation, the exact chemical composition of these interfacial foulants was subsequently extracted and analyzed. Extracellular polymeric substances (EPS) play a pivotal role in mediating the adhesion of microbial cells to membrane surfaces and the cohesion of the bio-cake matrix. To qualitatively map the distribution of these organic foulants, three-dimensional excitation–emission matrix (EEM) fluorescence spectroscopy was employed. Figure 6 presents the EEM spectra of the EPS extracted from the fouled membrane surfaces. Across all membrane samples, the spectra consistently exhibited distinct fluorescence peaks, with tryptophan-like and tyrosine-like proteins dominating the organic profile [32]. However, the fluorescence intensities varied drastically depending on the membrane architecture. The symmetric membrane displayed relatively weak fluorescence signals in the protein-like regions. In stark contrast, the asymmetric membranes exhibited remarkably amplified fluorescence intensities, with the signal strength of the tryptophan-like proteins escalating visibly as the separation layer thickness increased. This qualitative mapping indicates a highly selective enrichment of proteinaceous foulants at the highly retentive asymmetric interfaces.

**Figure 7:**
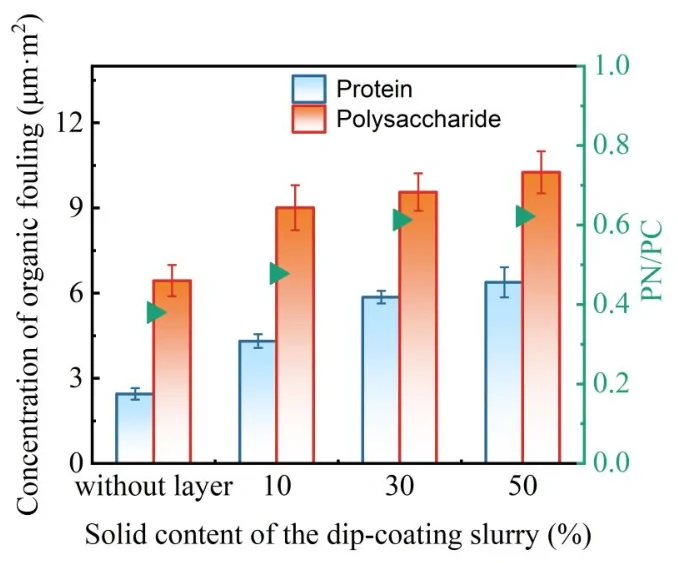
Concentrations of organic fouling (protein and polysaccharide) and the PN/PC ratio (green triangle) on the surfaces of membranes with different solid contents of the dip-coating slurry.

To quantitatively substantiate these spectroscopic observations, the absolute areal concentrations of proteins and polysaccharides within the interfacial EPS were determined (Figure 7). The quantitative data perfectly mirrored the EEM trends, revealing a fundamental shift in foulant composition. On the symmetric membrane, the accumulated protein concentration was relatively low (2.45 ± 0.20 μg·cm^−2^). Conversely, the deposition of the asymmetric separation layer exacerbated the organic accumulation, causing the interfacial protein concentration to surge dramatically to 4.31 ± 0.24 μg·cm^−2^, 5.86 ± 0.22 μg·cm^−2^, and 6.38 ± 0.53 μg·cm^−2^ as the separation layer thickness increased. Consequently, the protein-to-polysaccharide (PN/PS) ratio experienced a significant leap from 0.380 for the symmetric support to 0.622 for the thickest asymmetric variant.

This profound shift in the organic chemical profile provides a definitive molecular explanation for the macroscopic fouling behaviors observed earlier. Proteins within the EPS matrix are inherently more hydrophobic, complex, and cohesive than hydrophilic polysaccharides [33]. The elevated local hydrodynamic drag of the asymmetric separation layer forces these sticky proteinaceous substances to selectively concentrate at the membrane–water interface, thereby facilitating intense intermolecular cross-linking. This localized cross-linking transforms the accumulated bio-flocs into a highly recalcitrant, gel-like matrix. Consequently, this chemically sticky and physically dense protein-dominated cake layer perfectly accounts for the dramatic surge in the irreversible fouling resistance previously quantified in the macroscopic filtration analysis.

### 3.4. Microbial Community

To uncover the biological origins of the highly proteinaceous cake layer, high-throughput 16S rRNA gene sequencing was utilized to profile the microbial communities inhabiting the bulk sludge and the distinct membrane interfaces. Alpha diversity analysis (Table S4) revealed that the spatial architecture of the membranes exerted a strong selective pressure on microbial colonization. The symmetric membrane harbored the least diverse microbial community. However, the implementation of the asymmetric separation layer significantly increased the community richness and diversity. As the separation layer thickened, the Shannon diversity index and the total number of operational taxonomic units (OTUs) steadily increased, indicating that the elevated local hydrodynamic drag on the asymmetric surfaces created an extreme micro-environment that trapped a broader spectrum of microbial species.

**Figure 8:**
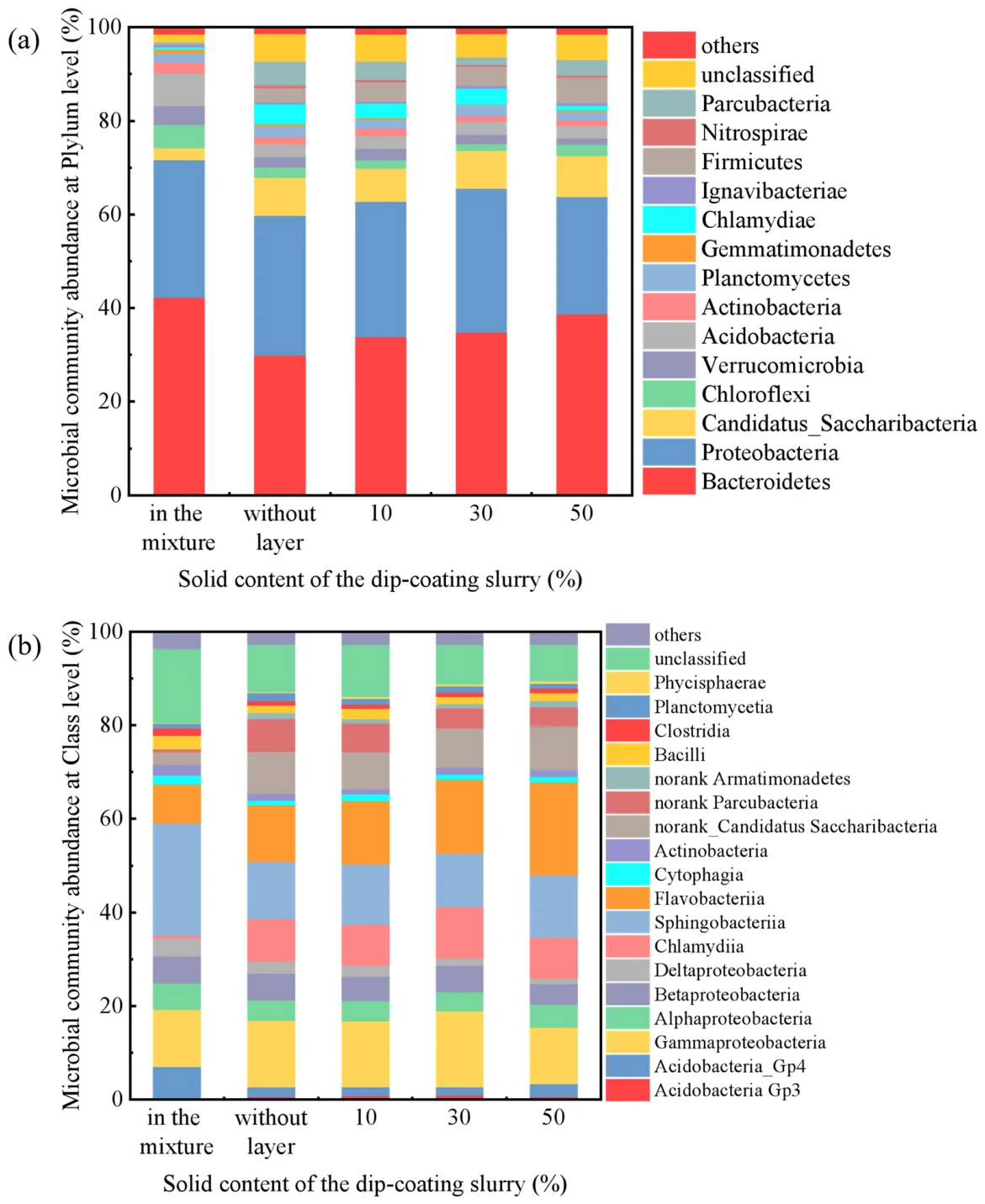
Relative abundance of microbial communities at the (**a**) phylum and (**b**) class levels in the bulk mixture and within the bio-cake layers on the fouled surfaces of different membranes.

The specific taxonomic composition further elucidates the biological mechanisms driving the severe irreversible fouling on the asymmetric membranes. As illustrated in the relative abundance profiles (Figure 8), Bacteroidetes and Proteobacteria dominated the interfacial bio-cake across all samples [34,35]. Notably, the physical refinement of the membrane surface induced a profound selective enrichment of specific fouling-associated taxa. At the phylum level, the relative abundance of Bacteroidetes, a phylum renowned for its prodigious capacity to secrete extracellular proteins and establish robust biofilms, surged from 30.38% on the symmetric membrane to 38.71% on the thickest asymmetric variant. Similarly, the abundance of Firmicutes, which are highly prone to surface attachment, noticeably escalated [36].

This architecture-associated shift in the interfacial microbial community, particularly the increase in Flavobacteriia from 11.99% to 19.97%, aligns with the known EPS-secreting and biofilm-forming capacities of these taxa and is consistent with the elevated protein accumulation observed in the EPS quantification [37,38], together indicating that membrane architecture influences the interfacial microbial composition in a manner that may contribute to proteinaceous fouling.

## 4. Conclusions

Low-cost and high-performance symmetric and asymmetric hollow flat-sheet alumina ceramic membranes were successfully fabricated, and their long-term fouling behaviors were systematically compared in municipal wastewater-treating MBRs. The results revealed that the membrane microstructure exerted a decisive influence on fouling resistance. Although the asymmetric architecture significantly enhanced solute rejection, it triggered a severe permeability decline, with pure water flux dropping to 2370 L/(m^2^·h·bar). Furthermore, its elevated local hydrodynamic drag promoted a massive accumulation of proteinaceous foulants, surging to 6.38 μg/cm^2^, and the selective enrichment of pioneer biofilm-forming bacteria, notably Flavobacteriia, which reached an abundance of up to 19.97%. This led to the accelerated formation of a dense, irreversible cake layer and a much faster TMP rising rate of 77.70 KPa/d. In stark contrast, the symmetric membrane with a 0.64 μm pore size, despite a slightly lower initial rejection capability, exhibited superior operational stability and a significantly lower fouling rate of 47.93 KPa/d owing to its homogeneous pore structure. The current assessment is based on a single 60-day operational cycle, and long-term performance under repeated chemical cleaning and flux recovery cycling remains to be evaluated. Future studies will incorporate multiple operational cycles with repeated chemical cleaning to assess long-term membrane lifespan and durability. These findings highlight a critical trade-off between selectivity and antifouling performance. This study was conducted using synthetic municipal wastewater under controlled laboratory conditions, and its applicability to full-scale MBR systems treating real municipal wastewater requires further validation through pilot-scale studies.

## Data Availability

The original contributions presented in this study are included in the article/Supplementary Material. Further inquiries can be directed to the corresponding author.

## Supplementary Materials

The following supporting information can be downloaded at: https://www.mdpi.com/article/10.3390/membranes16090297/s1, Table S1. Composition of the synthetic municipal wastewater used in the MBR; Table S2. Linear expressions of the Hermia model for constant-flux filtration; Table S3. Linear fitting results of ceramic membrane flux variation in MBRs; Table S4. Comparison of alpha diversity indices of microbial communities between the mixed liquor and the membrane; Figure S1. Particle size distributions of the α -alumina aggregate materials. (a) The 8 μm α-Al2O3 powder used in the symmetric support membrane; (b) the 1 μm α-Al2O3 powder used in the dip-coating slurry for the asymmetric separation layer; Figure S2. X-ray diffraction (XRD) patterns of the symmetric support membrane and the asymmetric separation layer; Figure S3. (a) Surface pore size distribution histogram of the asymmetric membrane. SEM images of the asymmetric membranes prepared with dip-coating slurry solid contents of (b, d) 30% and (c, e) 50%. (b) and (c) show the surface morphologies (with high-magnification insets), while (d) and (e) display the corresponding cross-sectional morphologies. The yellow dashed lines indicate the separation layers with thicknesses of 97 μm and 153 μm, respectively; Figure S4. Comparison of the mass loss rates of the symmetric and asymmetric membranes in strongly acidic (pH = 1) and strongly alkaline (pH = 13) solutions for 120 h; Figure S5. Three-dimensional surface topography images of (a) the symmetric support membrane and (b) the asymmetric separation layer. The scan area for both images is 5.0 μm × 5.0 μm. (c) Comparison of the root-mean-square (RMS) surface roughness between the asymmetric and symmetric membranes; Figure S6. Variation in water quality parameters in the feed water and permeates of different membranes during long-term continuous operation: (a) chemical oxygen demand (COD); (b) ammonia nitrogen (NH3-N); (c) total phosphorus (TP).
