# Supplementary material for: Structural Design of Ceramic Membranes to Mitigate Fouling in Membrane Bioreactors
Source: Membranes (Basel). 2026 Sep 10;16(9):297. doi: 10.3390/membranes16090297 (PMC13609629; doi:10.3390/membranes16090297)
Supplement: Supplementary file 1 [file membranes-16-00297-s001.zip › membranes-4516089-supplementary.pdf]

Text S1 Detailed Membrane Preparation Procedures

Symmetric Membrane Preparation:

Industrial-grade α-alumina (α-Al_2O_3) powders with a median particle size (d_{50}) ranging from 1.0 μm to 20.0 μm were utilized as the skeleton material to tailor the pore sizes. For the optimized symmetric support, 8 μm α-Al_2O_3 powder was uniformly mixed with 3 wt% kaolin, 2.5 wt% hydroxypropyl methylcellulose, and a -titania composite micro-powder. Additionally, 4 wt% soybean oil and 16-19 wt% deionized water were added to achieve a homogenous, extrudable plastic paste. The paste was vacuum-pugged, aged for 24 h at room temperature, and extruded through a customized die to form green hollow flat-sheet bodies (72×6 mm cross-section with 15 internal channels of 4×4 mm). After natural drying, the green bodies were sintered in air at 1550 °C for 4 h.

Asymmetric Membrane Preparation:

The asymmetric separation layers were applied onto the optimized symmetric supports via a dip-coating method. The coating slurry was prepared by dispersing 1 μm α-Al_2O_3 powder in deionized water, supplemented with ceramic dispersan, sodium bicarbonate, and sodium silicate to maintain pH and stability. The suspension was ball-milled for 48 h at 500 r/min. To obtain varying separation layer thicknesses , the solid content of the α-Al_2O_3 slurry was precisely adjusted to 10 wt%, 30 wt%, and 50 wt%, respectively. The support membranes were vertically immersed into the slurry for 30 s and withdrawn at a constant velocity of 10 cm/s. The wet membranes were dried in a climatic chamber (45 °C and 80% humidity) to prevent micro-cracking,

followed by a secondary calcination at 500 °C for 2 h to remove organic additives and consolidate the active layer.

Text S2. Mathematical Modeling of Membrane Fouling

Resistance-in-Series Model:

Based on Darcy's law, the total hydraulic resistance (R_t) during the filtration process was quantified and decoupled into specific fouling components using the following equation:

$$R_t = R_m + R_{pr} + R_{cr} + R_{ir} = \frac{\Delta P}{\mu \cdot J} \quad \text{(S1)}$$

Where R_m is the intrinsic membrane resistance (m^{-1}), R_{pr} is the physically reversible fouling resistance (m^{-1}) removed by hydraulic backwashing and surface rinsing, R_{cr} is the chemically reversible fouling resistance (m^{-1}) removed by sodium hypochlorite soaking, and R_{ir} is the irreversible fouling resistance (m^{-1}) that cannot be removed by standard cleaning protocols. ΔP is the transmembrane pressure (bar), μ is the dynamic viscosity of the permeate (Pa·s), and J is the permeation flux ($L \cdot m^{-2} \cdot h^{-1} \cdot bar^{-1}$).

Hermia's Filtration Model:

To elucidate the dominant fouling mechanisms during the constant-flux MBR operation, the TMP evolution data were fitted to the classical Hermia model, expressed by the general differential equation:

$$\frac{d^2t}{dV^2} = k\left(\frac{dt}{dV}\right)^n \quad \text{(S2)}$$

Where t is the filtration time, V is the cumulative permeate volume, k is a phenomenological constant, and n is a fouling index characterizing the specific fouling mechanism (n=2 for complete pore blocking, n=1.5 for standard pore blocking,

n=1 for intermediate pore blocking, and n=0 for cake layer filtration). The linear expressions for these four mechanisms are summarized in **Table S2**.

Text S3 EPS separating method by centrifugation and thermal extraction

25 mL of mixed liquid was firstly centrifuged at 4000 g for 5 min at 4 °C. The pellet left in the centrifuge tube was then re-suspended to its original volume by adding 0.05% NaCl solution. The suspension was sheared by a vortex mixer for 1 min; afterward, it was centrifuged at 4000 g for 10 min at 4 °C, and the supernatant was collected as LB-EPS. The sludge left in the tube was resuspended again to its original volume of 25 mL by adding 0.05% NaCl solution, and then it was put into a water bath at 60 °C for 30 min. It was centrifuged at 4000 g for 15 min at 4 °C, and the supernatant was labeled as TB-EPS.

Table S1 Composition of the synthetic municipal wastewater used in the MBR.

**Component**	**Concentration (mg·L^{-1})**
Glucose	450
NH_4Cl	95
KH_2PO_4	20
$MgSO_4$	50
$CaCl_2$	6.25
$FeCl_3·6H_2O$	1.85
Trace elements (Cu^{2+}, Mn^{2+}, Co^{2+})	< 1.5

Table S2 Linear expressions of the Hermia model for constant-flux filtration.

Fouling Mechanism	*n* value	Linear Expression
Complete pore blocking	2	$\ln(J^{-1}) = \ln(J_0^{-1}) + k_{cb}t$
Standard pore blocking	1.5	$J^{-0.5} = J_0^{-0.5} + k_s t$
Intermediate pore blocking	1	$J^{-1} = J_0^{-1} + k_i t$
Cake layer filtration	0	$J^{-2} = J_0^{-2} + k_{ef} t$

J and J_0 represent the specific flux at time t and initial time, respectively; k represents the corresponding kinetic constants.

Table S3 Linear fitting results of ceramic membrane flux variation in MBRs.

Membrane sample	Fouling Mechanism	*n* value	R^2
without layer	Complete pore blocking	2	0.978
10% solid content	Cake layer filtration	0	0.893
30% solid content	Cake layer filtration	0	0.903
50% solid content	Cake layer filtration	0	0.944

Table S4 Comparison of alpha diversity indices of microbial communities between the mixed liquor and on the membrane.

	microbial in the mixed liquor	microbial on the membrane			
		Solid content of the dip-coating slurry			
		without layer	10	30	50
Chao1	812.33	781.84	783.95	796.66	799.43
Shannon	5.63	4.53	4.68	4.75	4.88

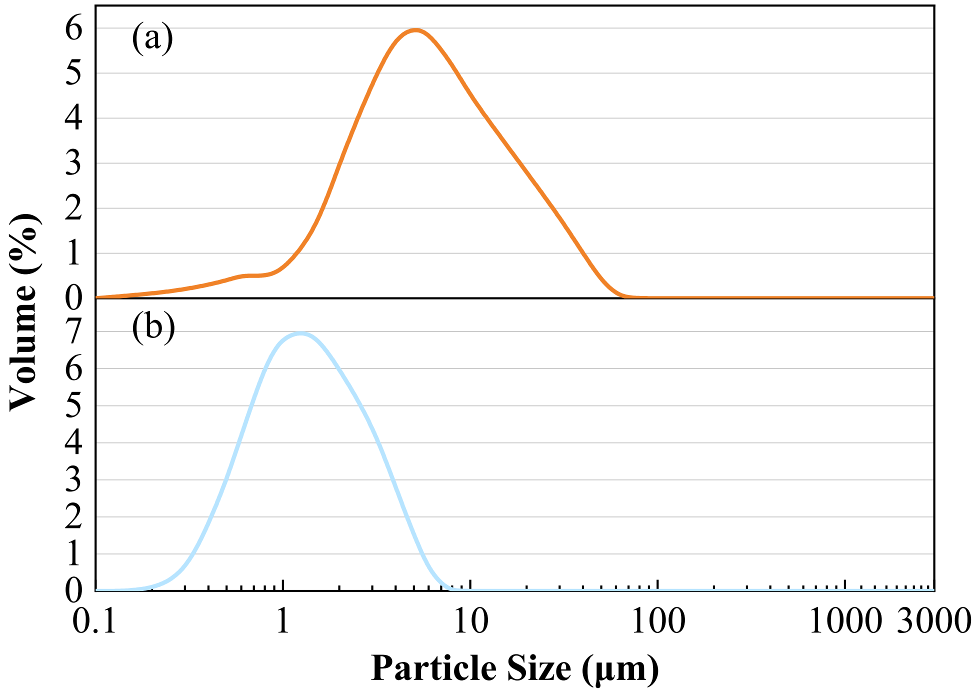

Figure S1. Particle size distributions of the α -alumina aggregate materials. (a) The 8 μm α-Al_2O_3 powder used in the symmetric support membrane, (b) The 1 μm α-Al_2O_3 powder used in the dip-coating slurry for the asymmetric separation layer.

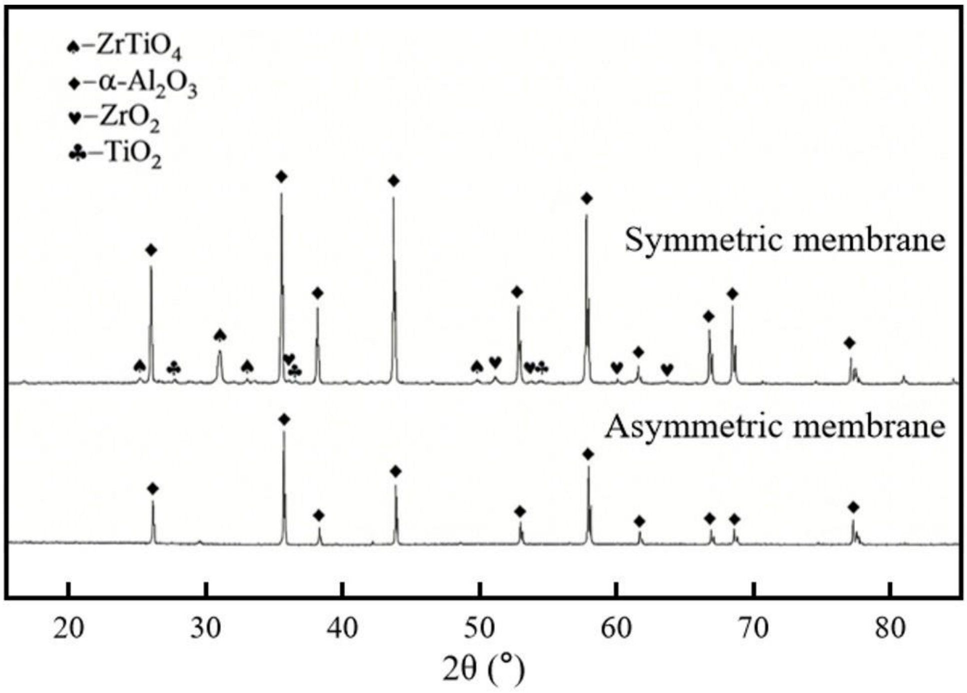

Figure S2 X-ray diffraction (XRD) patterns of the symmetric support membrane and the asymmetric separation layer.

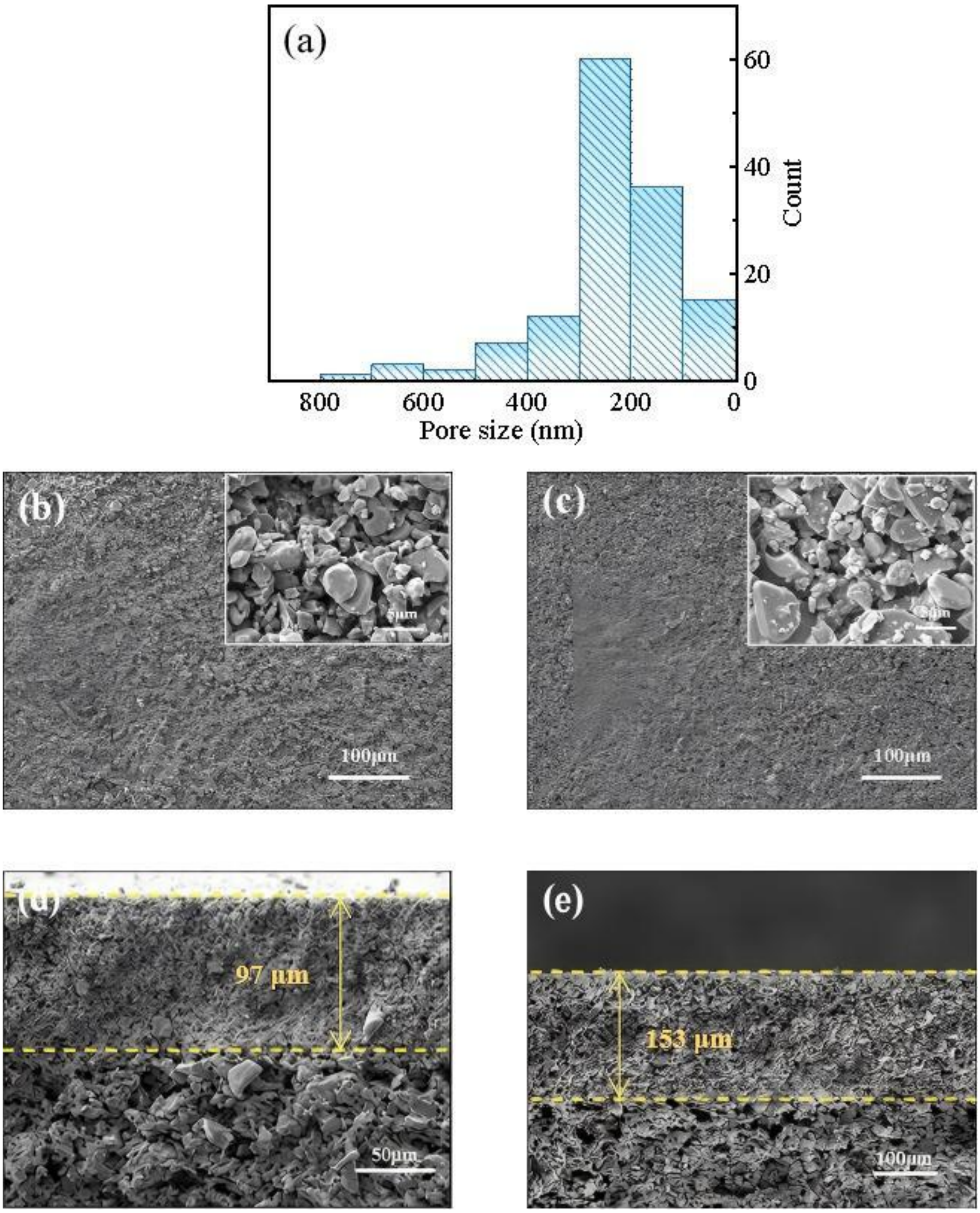

Figure S3 (a) Surface pore size distribution histogram of the asymmetric membrane. SEM images of the asymmetric membranes prepared with dip-coating slurry solid contents of (b, d) 30% and (c, e) 50%: (b) and (c) show the surface morphologies (with high-magnification insets), while (d) and (e) display the corresponding cross-sectional morphologies. The yellow dashed lines indicate the separation layers

with thicknesses of 97 μm and 153 μm, respectively.

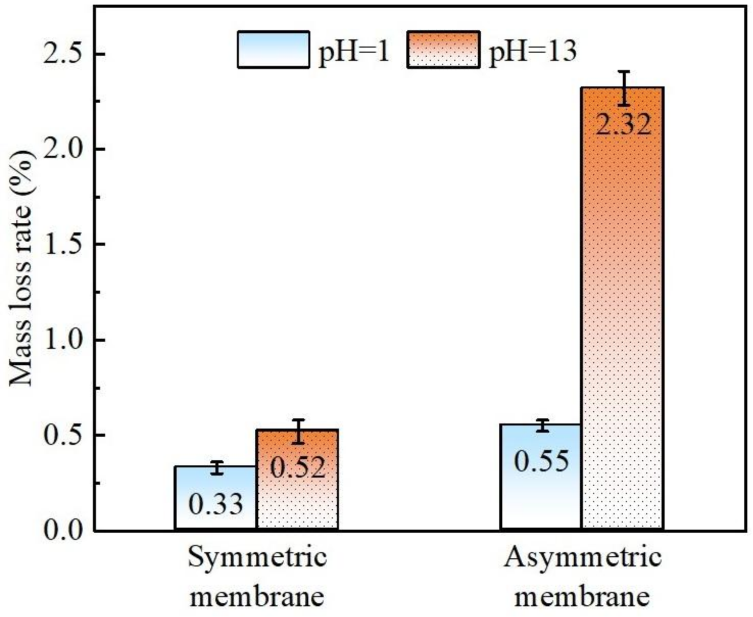

Figure S4 Comparison of the mass loss rates of the symmetric and asymmetric membranes in strongly acidic (pH = 1) and strongly alkaline (pH = 13) solutions for 120 h.

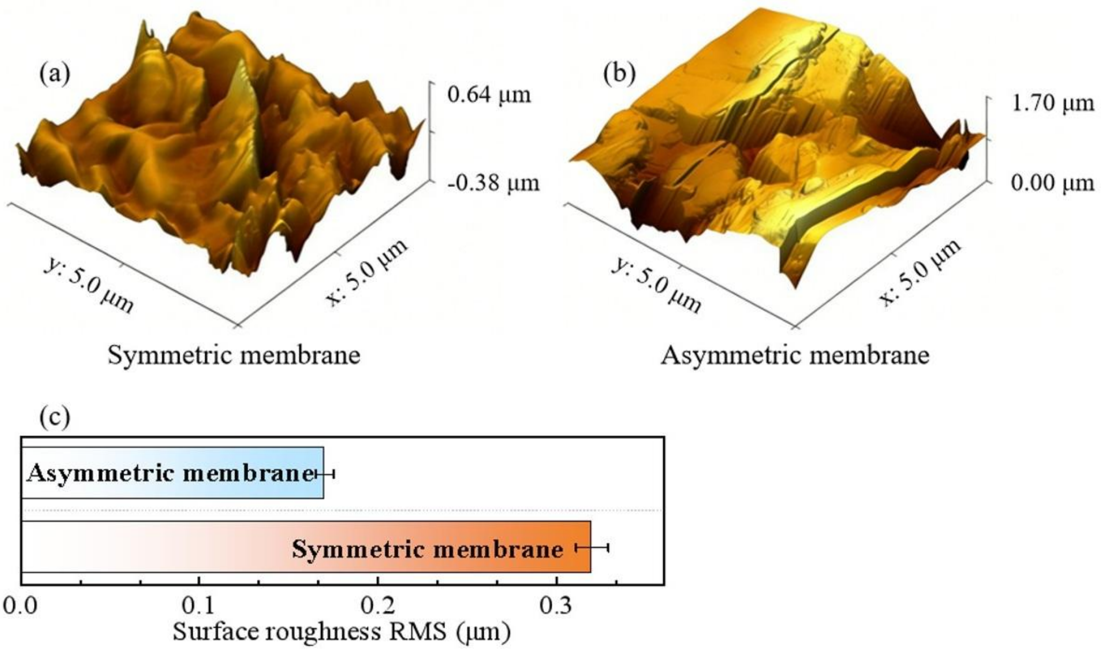

Figure S5 Three-dimensional surface topography images of (a) the symmetric support membrane and (b) the asymmetric separation layer. The scan area for both images is

5.0 µm × 5.0 µm. (c) Comparison of the root-mean-square (RMS) surface roughness between the asymmetric and symmetric membranes.

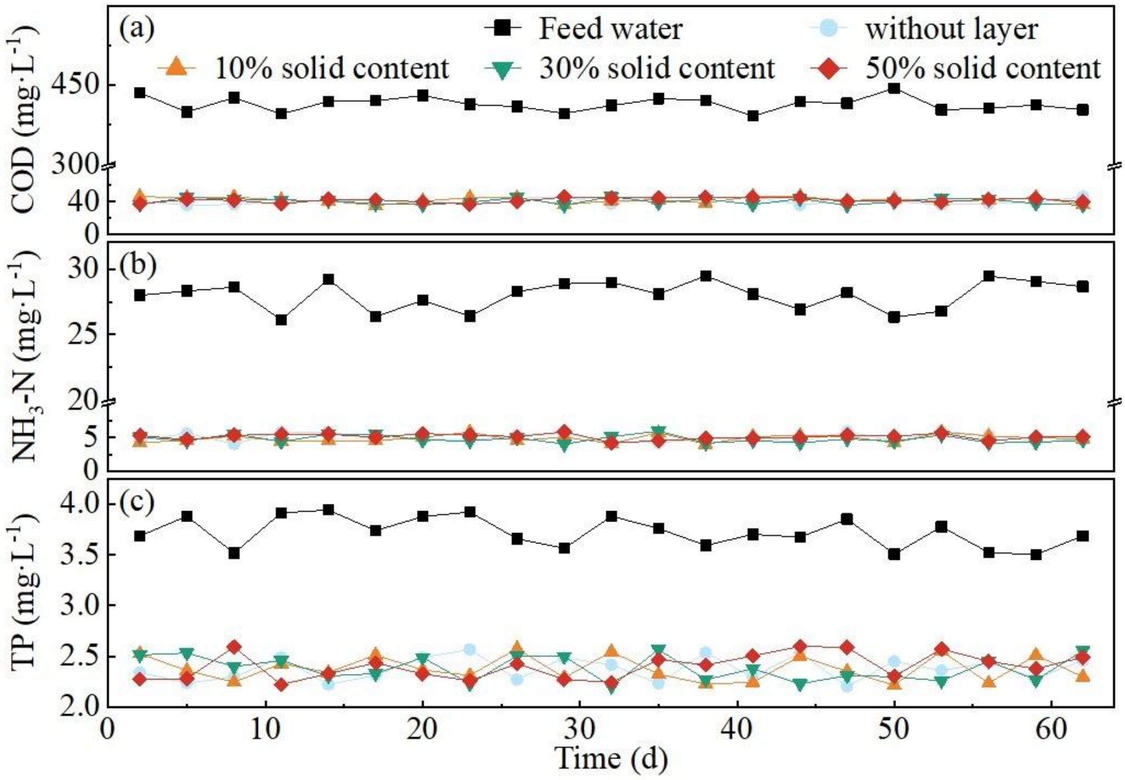

Figure S6 Variations of water quality parameters in the feed water and permeates of different membranes during long-term continuous operation: (a) chemical oxygen demand (COD); (b) ammonia nitrogen (NH_3-N); (c) total phosphorus (TP).
